# Addressing the Sense of School Belonging Among All Students? A Systematic Literature Review

**DOI:** 10.3390/ejihpe14110190

**Published:** 2024-11-12

**Authors:** Urška Štremfel, Klaudija Šterman Ivančič, Igor Peras

**Affiliations:** Educational Research Institute, Gerbičeva ulica 62, 1000 Ljubljana, Slovenia; klaudija.sterman@pei.si (K.Š.I.); igor.peras@pei.si (I.P.)

**Keywords:** sense of school belonging, predictors, bioecological model of human development, equity, systematic review

## Abstract

The sense of school belonging plays an important role in students’ academic, behavioural, and psychological outcomes. Based on a systematic review, following the PRISMA 2020 guidelines and examining 86 studies conducted between 1990 and February 2023, the article addresses two research questions: (a) what are the predictors of the sense of school belonging at the individual, micro, meso, exo, macro, and chrono levels of the bioecological model of human development; (b) do these predictors differ based on students’ individual characteristics, and if so, how. The findings reveal individual factors as important predictors of school belonging and indicate the lack of studies that take into consideration the interplay of different (micro, meso, exo, macro, chrono) levels in addressing the sense of school belonging. Considering the complexity and multi-factorial nature of the sense of school belonging, it calls upon further research, which would support the development of evidence-based interventions for fostering school belonging among different groups of students, particularly those who are at risk of feeling alienated from school, and thus promote equity in education.

## 1. Introduction

The need to belong is a fundamental human motivation [1]. Humans have a pervasive need to form and maintain at least a minimum quantity of lasting, positive, and significant relationships [2]. Given that school is a primary socialising unit and a relational community that offers opportunities for students to fulfil the need to belong [3], it is not surprising that a sense of school belonging has been at the forefront of educational and cross-sectional research (e.g., in the fields of psychology, sociology, and health-promotion) in recent decades.

### 1.1. Defining a Sense of School Belonging

Students spend a significant amount of time in school attending classes, forming relationships, socialising with peers, and participating in extra-curricular activities; they therefore tend to develop feelings about their school. These feelings are known as a sense of belonging to school—or school belonging. Students with a higher sense of school belonging have positive feelings towards their school, while students with a lower sense of school belonging feel alienated [4]. School belonging is a part of students’ subjective perception of their place in the school’s social environment; it has also been referred to as psychological membership [5]. Various terminology has been used to describe the construct of school belonging, including school bonding, school connectedness, school attachment, school identification, belongingness, a sense of community, school engagement, and school involvement [6,7,8]. Even if the terminology used to describe school belonging varies, the underlying themes (i.e., students having an emotional attachment to others, having a place within their school, and having a sense of inclusion) noted in the definitions remain the same.

To provide a broad overview of studies assessing the sense of school belonging, the present review was guided by the definition proposed by Goodenow and Grady [9] who defined school belonging as the extent to which students feel personally accepted, respected, included, and supported by people, especially teachers and other adults, in the school’s social environment. As argued by Allen et al. [10], this definition emphasises the multiple features of school belonging for students, as well as the broader socio-ecological context of peers, students, and teachers within the school environment. The use of Goodenow and Grady’s [9] definition is reasonable and justified given the aim of the present systematic review—to identify as many predictors of school belonging as possible and classify them according to the Bronfenbrenner [11] bioecological model of human development.

### 1.2. A Framework for Assessing Predictors of School Belonging

According to the bioecological model [11], a person’s environment consists of several levels that range from the immediate micro-system with which the person has direct contact to the larger macro-system, which is composed of the structural and institutional arrangements of the country in which the person resides, as well as changes over time in the person and environment. The Bronfenbrenner model helps understand the multi-layered social contexts that affect the sense of school belonging [6,10,12,13]. According to Allen et al. [10], the sense of school belonging is influenced by individual, relational, and organisational factors inside a broader school setting and within a certain political, geographical, and cultural landscape unique to that setting. The Bronfenbrenner socio-ecological model therefore posits that a sense of school belonging may result from a combination of factors that stretch across multiple interrelated layers of the social environment, not simply from individual-level factors [14,15].

The individual level refers to the individual student, and the predictor variables at this level represent the student characteristics that contribute to his or her sense of school belonging (e.g., demographic characteristics such as gender, age, minority status, academic achievement, academic self-regulation, future aspirations, depressive symptoms, anxiety, and other personal characteristics) [16,17,18,19,20,21]. The micro level showcases the importance of close relationships for a student’s sense of school belonging. Relationships with parents, peers, and teachers are at the forefront of this level (e.g., family support for learning, parent–student relationships, positive relationships among students and teachers, and academic support shown by teachers and peers) [19,20,22]. The meso level includes the broader school environment, including school processes, practices, pedagogy, and policies (e.g., students’ participation in extracurricular activities, school policy measures, and support for staff’s professional development). The exo level refers to the broader context in which the school is situated (e.g., the school neighbourhood). The macro level refers to even broader aspects, such as public policies and legislation at the national level (e.g., laws and strategies). The chrono level refers to changes over time in the person and environment (e.g., the sense of school belonging measured at different time points).

### 1.3. Research Gaps

Considerable amounts of research, including some meta-studies [23] and systematic reviews [24,25], have examined the sense of school belonging as an independent variable predicting specific positive and negative academic, behavioural, and psychological (socio-emotional) outcomes among students. Less, but still significant, scholarly attention has been devoted to different predictors of the sense of school belonging [26]. Because of the different approaches to investigating school belonging, however, the findings are fragmented across the literature [27,28], and to date, little is known about the actual predictors that influence students’ sense of school belonging [27,29,30,31,32,33], and even less is known about the sense of school belonging among different groups of students. A lack of consideration of the predictors of the sense of school belonging has limited the understanding of this important educational construct and therefore presents an open challenge for educational research, which this systematic review aims to adress.

A systematic review of the fragmented literature in the field should increase our understanding of the possible array of sources that influence the sense of school belonging. Classifying those sources according to the bioecological model of human development [11] could help us assess how various systems contribute to the students’ sense of school belonging, especially when their individual characteristics are taken into account. Taking into consideration the revealed positive outcomes of students’ high sense of school belonging and the negative outcomes of a low sense of school belonging makes research progress in the field necessary and crucial. Knowing and understanding the predictors of school belonging is important not only for building theories of students’ sense of school belonging [16] but also for designing evidence-based policy interventions and educational practices for its promotion [34]. This systematic review builds on an existing meta-study [6] and systematic review [35] in several ways to contribute to a thorough and in-depth understanding of the predictors of the sense of school belonging.

The sense of school belonging is a developing research field, and this review thus takes the latest research into account. It also involves studies that researched the sense of school belonging among students aged 6–19 years old, while existing reviews have focused on adolescent students (12–19 years old). As Quinn and Oldmeadow [36] pointed out, one cannot ignore the fact that a sense of belonging is important for children of all ages. Elementary school is the foundational period for students’ formal education, so students’ experiences in elementary school directly affect their subsequent school development [37,38]. The Progress in International Reading Study (PIRLS) and Trends in International Mathematics and Science Study (TIMSS) measured the achievement of fourth-grade students, and some individual studies [28,39,40] have also confirmed this importance. Existing evidence, however, suggests that different factors may influence the sense of school belonging at different year levels [28].

In addition to quantitative studies, qualitative studies are also included in the systematic review. This may extend the possible array of factors of school belonging, which may have been overlooked in quantitative studies. Although Bronfenbrenner’s [11] bioecological model of human development has already been recognised as beneficial for understanding the different predictors at different levels (individual, micro, meso, exo, macro, and chrono) that affect the sense of school belonging [6], this systematic review builds on these findings by not limiting itself to certain pre-selected predictors and by taking the interplay of individual-level and other bioecological-level predictors into account. This review thus considers that existing individual studies have revealed different groups of students experiencing different levels of the sense of school belonging [16,17] and examines whether and how the predictors of the sense of school belonging differ among different groups of students. These findings are then critically discussed through the lens of equity in education as an important educational goal, which should enable all students to develop their potential [41].

### 1.4. Aims and Research Questions

The present systematic review aims to identify the predictors of school belonging by examining studies where a sense of school belonging was examined as a dependent variable or outcome. Using Bronfenbrenner’s ecological theory, we systematically classify predictors of school belonging at different levels of the students’ environment. Recognising that a sense of school belonging is the result of the interplay of individual and wider environmental factors, this systematic review also aims to clarify which environmental factors are important for fostering the sense of school belonging among different groups of students. Additionally, we critically examine the findings from the perspective of equity in education. Based on these notions, this systematic review was driven by the following research questions: (a) What are the predictors of a sense of school belonging at the individual, micro, meso, exo, macro, and chrono levels; (b) Do these predictors differ based on students’ age, gender, minority status, and academic achievement and/or other students’ individual characteristics, and if so, how?

## 2. Method

### 2.1. Literature Search—Phase 1 (Database Search)

The present systematic review used the latest checklist of the Preferred Reporting Items for Systematic Review and Meta-Analysis (PRISMA) statement [42]. We employed a systematic literature review methodology to provide a comprehensive and rigorous examination of the existing research on predictors of the sense of school belonging. Our review followed the updated PRISMA 2020 checklist [42], addressing all relevant criteria, except result analyses, as this systematic review did not include meta-analysis. The review and its protocol were not previously registered.

The following search string was used to identify articles for the systematic literature review: belong or connectedness or relatedness or membership or sense belong*, predictor or factor, and school. The same search string was applied consistently across all corresponding databases.

The following five databases were used to identify relevant journal articles in March of 2023: Web of Science, PsycARTICLES, ERIC, Scopus, and SOC INDEX. The databases were selected to capture the multidisciplinary nature of the sense of school belonging, a construct that spans various research fields such as psychology and educational sciences. This selection aligns with the interdisciplinary scope of the topic, ensuring access to a comprehensive range of peer-reviewed journal articles.

Inclusion and exclusion criteria are summarised in Table 1. To be included in the review, articles needed to meet all specified inclusion criteria. Additional details on these criteria, along with examples of excluded studies, are provided below:

(1) Sense of school belonging was operationalised as the sense of school belonging or school belonging or defined in a way that mirrored the definition of Goodenow and Grady [9]. Specifically, studies researching school belonging according to this definition typically employ the Psychological Sense of School Membership Scale (PSSM) but also other related constructs (i.e., school connectedness as measured by Sampasa-Kanyinga et al. [43], peer relatedness as measured by Mikami et al. [44], school bonding as measured by Oelsner et al. [45]).
(2) Sense of school belonging (or any related construct) was measured as a dependent variable or outcome. Therefore, studies in which the construct was not measured as the outcome or dependent variable were omitted.
(3) All possible variables that contribute to a sense of school belonging were included to be later organised into groups and presented as results at the individual, micro, meso, exo, macro, and chrono levels. Studies were excluded if coding predictors or themes related to the sense of school belonging was not feasible.
(4) Participants in the study were between 6 and 19 years of age, which is the typical age range for students in primary and secondary school. In other words, studies focusing on college students or school belonging in the college setting were excluded. This was achieved by reviewing the sample sections of each article’s methodology; studies were excluded if they specifically mentioned college students or included age ranges corresponding to college-aged individuals or older.
(5) The article describing the study was written in the English language but not geographically limited. Studies not written in the English language were excluded.
(6) The peer-reviewed articles included in this review were published between 1990 and 2023, with search criteria set to exclude studies published prior to 1990. This timeframe was selected because research on the sense of school belonging emerged around this period, marking the initial development of studies on the construct [9,46]. Studies outside the specified timeframe were excluded.

**Figure 1:**
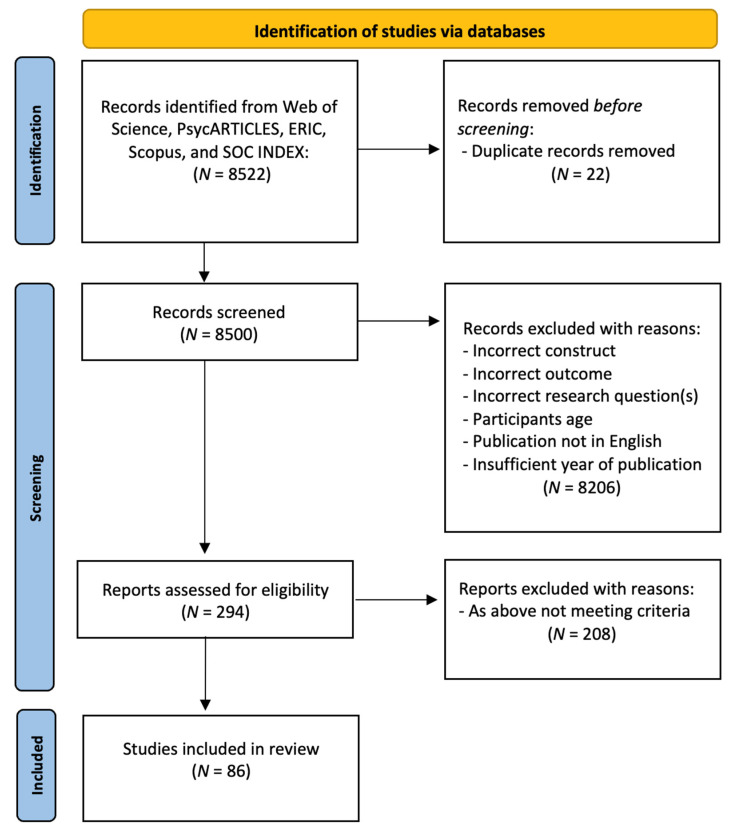
PRISMA 2020 flow diagram for new systematic reviews. Figure created based on PRISMA 2020 template [42].

Figure 1 depicts the steps of the systematic review methodology used in the present study. In the identification stage, the search yielded a total of 8522 titles, of which 8500 remained after duplicates were excluded. Duplicates were removed manually after all titles were entered into a Microsoft Excel spreadsheet. In the screening stage, the titles and abstracts of all 8500 articles were screened using a set of predetermined selection criteria (Table 1) to obtain an initial draft list of eligible studies for inclusion in the systematic review. Based on the initial screening of titles and abstracts, we selected 294 studies that potentially met our inclusion criteria (studies were excluded if there was explicit evidence that any inclusion criteria had been breached). These studies were all thoroughly read by one author and assessed against the inclusion criteria (Table 1) once more. In the third step, based on a complete reading of all of the articles, 86 articles were selected for inclusion in this systematic review. Studies were excluded if they did not fit each of the inclusion criteria defined. Due to the large number of studies that met the criteria for inclusion in the systematic review after the initial database search, we opted not to expand our search (i.e., checking the reference lists of identified studies or further identifying studies based on a secondary search of the databases).

### 2.2. Information Retrieval and Coding Process

To collect the descriptive information on all included studies, we coded the selected studies in a Microsoft Excel spreadsheet. A coding scheme detailing what to extract from each study was developed based on the initial research questions.

The following information was coded: authors of the study, year of publication, journal name, location (country of data collection), description of the sample (number and age of students, school level, number of schools), dependent variable (operationalisation of the school belonging construct), identified predictors, description of the study focusing on how the data were collected, and data analysis method. Data were extracted and coded by one author, with any issues resolved through collaborative discussions with the other authors to ensure accuracy. As a single author performed all coding, reliability statistics (e.g., kappa coefficient) were not calculated.

Upon completing the extraction and coding process, a comprehensive table of all studies and predictors was compiled in Microsoft Excel. One of the authors grouped the predictors into themes and classified them based on the levels of Bronfenbrenner’s bioecological model. Two remaining authors independently checked the predictors and their classification. Discrepancies were resolved by conversation until an agreement was reached.

## 3. Results

### 3.1. Characteristics of Reviewed Studies

A total of 86 studies were reviewed in the present systematic review. A summary of the included studies is presented in Table S1 in Supplementary Materials. Quantitative predictors are reported from 77 studies (which reported results based on multiple regression, structural equation modeling, growth curve modeling, path analysis, and hierarchical linear modeling), and qualitative factors are reported from nine studies (which reported results based on qualitative analysis, thematic analysis, or interpretative phenomenological analysis). Only statistically significant predictors from quantitative studies and factors presented as contributing to students’ sense of school belonging from qualitative studies have been included in the present systematic review. Studies were published between 1998 and February 2023. Data were collected between 1988 and 2022. Most of the studies reviewed (81 of 86) were conducted in individual countries, and the majority of these studies were conducted in the United States of America (38 studies), 14 studies were conducted in Europe, 13 studies in Australia and New Zealand, 12 studies in Asia, three in Canada, and one in Peru. The remaining five studies used data from multiple countries collected in international large-scale assessment studies (e.g., Programme for International Student Assessment, PISA).

### 3.2. Studies (Not) Identifying Predictors at Different Bioecological Levels

**Figure 2:**
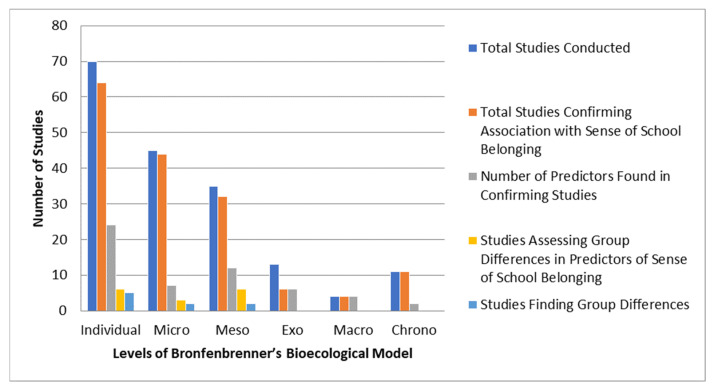
Distribution of Studies and Findings Across Different Levels of Bronfenbrenner’s Bioecological System.

This section (Figure 2) presents the number of studies that took predictors at different bioecological levels into account and the number of predictors identified. These data may explain the small/huge number of predictors at each level identified and the number of predictors for which the moderate to strong effect was not confirmed, and they indicate research gaps in the field.

The systematic review revealed that the greatest number of predictors of the sense of school belonging, which have a moderate to large effect, was identified at the individual level (24 predictors) by the 70 studies identified. At the micro level, seven predictors were identified in 45 of the studies analysed. At the meso level, 12 predictors were identified, although all of the studies analysed (35) did not consistently reveal the particular factor/predictor as having a moderate or strong effect on the sense of school belonging. At the exo level, six predictors were identified by 13 studies. At the macro level, four predictors were identified by four studies analysed. At the chrono level, all studies analysed (11) identified changes in the sense of school belonging over time. In the next section, we present the predictors identified in the studies included in the review in more detail.

### 3.3. Predictors of School Belonging from the Studies Reviewed

In examining the predictors of a sense of school belonging, we systematically categorised findings from the 86 studies reviewed, aligning each identified predictor with Bronfenbrenner’s bioecological model of human development to capture the contextual levels influencing students’ sense of school belonging. We classified the identified predictors into themes (Table 2). The themes were then further classified according to the individual, micro, meso, exo, macro, and chrono levels of Bronfenbrenner’s bioecological model of human development. To better understand the association of a specific predictor with the sense of school belonging, effects were interpreted as in the work done by Bardach & Klassen [47]. Thus, in quantitative studies, we did not simply focus on whether a predictor was significant or not; if sufficient data were available on the association (correlation) between predictor and sense of school belonging, we interpreted the magnitude of the association. Effects were interpreted in line with Cohen’s [48] recommendations: values over 0.10, 0.30, and 0.50 were interpreted as small, medium, and large, respectively. Values below 0.10 were defined as trivial.

In all of the studies (Table 2) where the associations were confirmed, the predictors on the individual proved to be significant but mostly had a small to moderate effect in explaining the sense of school belonging. Predictors that proved to have the strongest but still moderate positive and negative effects on the individual level were student’s academic behaviour (e.g., active participation, doing homework, academic engagement, prosocial behaviour, skipping school, nonattendance, being unprepared for class) [19,20,21,49,50,51,52,53], problematic behaviour (e.g., deviant behaviour, conduct problems, delinquency) [45,54,55], student’s internalising (e.g., depression and anxiety) [32,50,56,57,58,59], externalising difficulties (e.g., sleep problems) [32,50,60], student’s future and educational expectations [21,61], well-being (e.g., general health, school satisfaction) [16,40,62,63], and social goals (e.g., social affiliation goals for school and social development goals) [28,64,65].

On a micro level, the predictor that proved to have the largest effect in explaining school belonging was the student’s relationship with teachers (e.g., connectedness to teachers, teacher support, teacher likeability) [20,33,66,67,68,69,70,71,72,73,74,75,76,77].

Fewer studies focused on identifying the predictors of school belonging on the meso, exo, macro, and chrono levels. In all of the studies where such associations were confirmed, the predictors proved to be significant, but there were only two predictors that proved to have a large effect in explaining school belonging: positive classroom climate [16,19,20,78] and school support practices (e.g., school guidance, support services, priority for pastoral care, support of special educational needs) [20,33,79,80,81].

On an exo level, six studies [52,82,83,84,85,86] were identified. In all of the studies, the associations between the mentioned predictors and school belonging proved to be significant, but it was not possible to identify the effect sizes for the predictors from the methodology used. On a macro level, four studies [14,67,69,87] were identified. In all of the studies, the associations between the mentioned predictors and school belonging proved to be significant, but here, too, it was not possible to identify the effect sizes of the predictors from the methodology used. On a chrono level, we identified 11 studies [15,21,44,55,60,88,89,90,91,92,93] that focused on longitudinal approaches to examining students’ sense of belonging, and all of the studies confirmed significant changes to the sense of school belonging over time.

Our review of 86 studies reveals that predictors of a sense of school belonging vary by Bronfenbrenner’s model levels: individual predictors showed small to moderate effects, teacher relationships had strong micro-level effects, and classroom climate and school support practices were key at the meso level. Limited studies at the exo, macro, and chrono levels showed significant associations, with chrono-level studies indicating changes over time. It is also important to note that this review does not consider the interrelatedness of predictors at different levels of the bioecological model of human development, as, for example, individual behaviour, teacher-student relationships, and classroom climate collectively shape students’ sense of school belonging.

### 3.4. Predictors of the Sense of School Belonging of Different Groups of Students

As explained in Section 3.2, few of the identified studies examined the predictors of the sense of school belonging among different groups of students or noted that these predictors differ among particular groups of students (grade level, gender, and immigrant status). In Table 3, the findings of the systematic review of those studies are presented.

Some studies (Table 3) included in the systematic review reported differences in the predictors of different groups of students (i.e., differences in the valence or significance of a given predictor). On the individual level, studies [16,17,60,87] mostly found predictors to differ by gender (e.g., a predictor was significant for one gender and not the other). Aerts et al. [17] reported that an arts educational track was a positive predictor for girls but not for boys; a technical track was found to be a negative predictor for boys and not for girls. Sexual orientation was a negative predictor for girls but not for boys [17]. Azagba et al. [87] found not living with both parents to be a negative predictor for girls but not for boys. Externalising difficulties (e.g., sleep problems) were found to be a negative predictor for boys but not for girls [60]. In addition, Ma [16] and Sampasa-Kanyinga et al. [43] reported that individual-level predictors differed based on students’ grade level or age. Ma [16] found socioeconomic status (SES) to be a positive predictor for Grade 8 students but not for those in Grade 6. Academic achievement was reported as a negative predictor for Grade 6 students and a positive predictor for Grade 8 [16]. Sampasa-Kanyinga et al. [43] reported age to be a negative predictor for high school students but not for middle school students.

On the micro level, predictors differed based on gender [77] and immigrant status [94]. Uslu and Gizir [77] found parental involvement at school to be a positive predictor for boys but not for girls and parental involvement at home to be a positive predictor for girls but not for boys. He and Fischer [94] found teaching practices to differ as predictors for different groups of students. Soft grading was a positive predictor for immigrant students in Italy and a negative predictor for non-immigrant students. In Germany, soft grading was a negative predictor only for immigrant students but not for non-immigrant students. Hard grading practices were found to be a positive predictor for non-immigrant students in Spain and Italy but not for immigrant students in these two countries. Hard grading practices were also a negative predictor for immigrant students in Germany but not for non-immigrant students.

On the meso level, predictors differed based on students’ age [16]. Classroom climate (i.e., academic pressure) was found to be a predictor for 6th grade students but not 8th grade students [16]. Ma [16] also found the school disciplinary practices (i.e., the disciplinary climate) to be a predictor of Grade 8 students but not Grade 6. No studies assessed the differences of groups of students based on their academic achievement (i.e., low achievement vs. high achievement). As indicated in Table 3, no studies were identified for predictors at the exo, macro, and chrono levels.

To summarise, the present review reveals that predictors of a sense of school belonging vary by gender, grade level, and immigrant status. For example, individual predictors like arts education and SES impact gender and grade differently, while micro-level influences such as parental involvement and grading vary by gender and immigrant status. Meso-level predictors, including classroom climate and disciplinary practices, differ by age. No group-difference studies were identified for exo-, macro-, or chrono-level predictors, nor studies that would assess the differences in the sense of school belonging based on students’ academic achievement.

## 4. Discussion

The present review indicates that the predictors at the individual level of the Bronfenbrenner bioecological system have received the most research attention, followed by predictors at the micro and meso levels. Furthermore, there also appears to be a lack of studies researching the interplay of individual and different environmental (micro, meso, exo, macro) factors, which would clearly indicate the predictors of the sense of school belonging among different groups of students.

Furthermore, we can conclude that predictors of students’ sense of school belonging vary across different levels of Bronfenbrenner’s bioecological model of human development, with most identified at the individual level, showing small to moderate effects. Teacher relationships on the micro level and classroom climate and school support practices on the meso level also proved to be significant. However, few studies explore interactions across different levels or account for factors like gender, grade level, and immigrant status, underscoring a gap in research on how these contexts interrelate. Emphasising predictors at multiple levels could support vulnerable student groups and promote equity in education.

To ensure equity in education, it is important that each individual’s needs are met and that students can cultivate their potential [41]. Taking into consideration some of the findings from previous individual studies, which indicated that some groups of students (e.g., boys, low SES students, low-achieving students, students with immigrant and minority backgrounds) express a lower sense of school belonging than their peers [16,17,95], along with other research findings that indicate school belonging is a predictor of several academic, behavioural, and psychological outcomes [23], a sense of school belonging can be understood as an important measure for minimising students’ barriers to success, maximising their potential, and thus promoting equity in education. Understanding equity as an opportunity to enable all students to develop their potential while respecting their differences and different needs calls for the development of positive school-level (Bronfenbrenner meso level) predictors that will enable each student to feel accepted and satisfy his/her basic psychological need to belong.

The limited number of such studies not only limits the achievement of the aims of this systematic review and our ability to answer the second research question—“Do predictors of the sense of school belonging differ based on students’ age, gender, minority status, academic achievement, and/or individual characteristics, and if so, how?”—but, more importantly, it also provides an important obstacle in planning evidence-based interventions for fostering school belonging among different groups of students, particularly those who are at risk of feeling alienated from school. Although there is evidence in favour of universal approaches that target all students (e.g., the whole-school approach) [34], Berryman and Elley [96] (pp. 989–990) noted that, “when the notion of belonging at school has been posited as having the same meaning and influence on educational experiences for all students then we are in danger of missing or trivializing the experiences for the marginalized, the othered or the alienated”. Furthermore, Cornelius-White [97] highlighted the relatedness of student-centred teacher variables and positive student outcomes, while DeNicolo et al. [98] exposed the importance of “humanizing pedagogies that honour students’ wholeness, acknowledge the intersectionality they embody, and recenter students’ lives in teaching and learning”. This means continuously engaging in assessments of all student needs and making necessary adjustments to the interventions, either to meet specific developmental needs at a particular grade level [80], needs originating from an immigrant background [86], or any other student individual characteristic.

The findings of this review highlight the importance of personal and psychological support within schools to enhance students’ sense of belonging. Fostering teacher–student relationships and implementing strategies that support mental health, engagement, and positive behaviour are essential. Policies that promote teacher training in social–emotional skills, supportive practices, and community engagement can further strengthen school belonging by cultivating a positive classroom climate and linking students with family and community resources [6,99].

### 4.1. Limitations

Before any conclusions can be drawn, it is important to recognise the limitations of this systematic review. First, most of the studies included in the systematic review were based on correlations or models that depended on the associations between variables. This means that although we are talking about predictors of school belonging, the research designs of most of the included studies did not take causality into account, as there were no experiments present. Therefore, we cannot fully assess the influence of the predictors on school belonging, as this is limited by the research methodology in the reviewed studies.

Second, as we gathered a sufficient quantity of studies for the review through the database search, we opted not to conduct a backward citations search (e.g., searching the reference lists of already included articles). There is thus a possibility that we have missed relevant studies due to our methodology.

By considering the sense of school belonging among different groups of students, the article neglects the intersectionality of these particular groups’ identities and their need to belong (see also [98]), as this is beyond the scope of the present article. However, this review exposes that the different needs of students should be considered when addressing their sense of school belonging.

The concentration of research in the United States, with limited representation from other regions, suggests that findings on the sense of school belonging may reflect cultural or systemic educational factors specific to Western contexts. This geographical distribution implies a potential gap in understanding a sense of school belonging in underrepresented regions, highlighting the importance of expanding research in diverse cultural and educational contexts to inform a more global understanding of factors that influence the sense of school belonging.

### 4.2. Recommendations for Future Research

To address the limitations of the findings in the review, future studies should incorporate longitudinal designs or experimental approaches that can better capture changes over time and identify cause–effect relationships in students’ sense of school belonging. Incorporating intersectionality into future research could also significantly enrich our understanding of the sense of school belonging by recognising the complexity of student identities. Examining how intersecting identities such as race, gender, and socioeconomic status shape students’ experiences could reveal nuanced insights into the factors influencing their sense of school belonging.

As the study highlights the importance of personal and relational support systems within schools, emphasising teacher–student relationships and inclusive school policies as key to fostering a sense of belonging, by addressing these diverse predictors at multiple levels, future research can build a more comprehensive, equity-centred foundation for supporting students’ sense of school belonging. Given the significant environmental changes (chrono level) brought about by the COVID-19 pandemic, which disrupted schooling in recent years, it should be regarded as an important factor influencing the sense of school belonging [100]. Future research should focus on identifying whether and how the predictors of school belonging changed during and after the pandemic.

## 5. Conclusions

The results of study reveal individual factors (e.g., age, gender, academic achievement, educational track, socioeconomic status) as important predictors of sense of school belonging and demonstrate the lack of studies that take into consideration the interplay of different (micro, meso, exo, macro, and chrono) levels in addressing the sense of school belonging, namely how relationships with parents, peers, and teachers (micro level), school processes, practices, pedagogy, and policies (meso level), school neighbourhood (exo level), public policies and legislation at the national level (macro level), and changes over time in the person and environment (chrono level) are related to the sense of school belonging of students with different individual characteristics.

The complex and multi-factorial nature of the construct of a sense of school belonging [101] revealed that fostering school belonging should be understood as a reciprocal process in which individual characteristics as well as environmental experiences and social processes may have an influence. Fostering the sense of school belonging for all students, particularly for vulnerable students, should be understood as an important measure for promoting equity in education, as school belonging is a predictor of several academic, behavioural, and psychological outcomes [23]. Further research on micro-, meso-, exo-, macro-, and chrono-level factors, as well as their interplay with individual factors, should be promoted to support evidence-based interventions in the field (see also [6]). The meso-level factors, which involve several school-level factors over which schools have control, and their relationship with individual-level factors, should be given particular consideration when treating the school as the main socialising unit which promotes the sense of school belonging, as well as the institution that plays an important role in promoting social equity. As such, the sense of school belonging not only functions as a privilege that adheres to other systemic privileges but as a right available to all students [102].

## Figures and Tables

**Table 1:** The criteria for inclusion and exclusion in the systematic literature search.

	Inclusion Criteria	Exclusion Criteria
Construct	Sense of school belonging or similar construct mirroring the definition by Goodenow and Grady [9]	Construct in the study is not sense of school belonging
Outcome	Sense of school belonging was studied as an outcome in the study	Sense of school belonging is not an outcome
Research question	Predictors or themes of sense of school belonging are assessed	Research question is not related to predictors or themes
Participants	Participants are limited to primary and secondary school students (age range: 6 to 19 years)	Participants do not fall within age range
Language	English	Not English
Publication	Peer reviewed, published between 1990 and 2023	Outside defined range

**Table 2:** Identified Significant Positive and Negative Predictors of Students’ Sense of School Belonging at Different Levels of Bronfenbrenner’s Bioecological Model of Human Development.

Level	Findings	
	Significant Positive Predictors	Significant Negative Predictors
Individual	Age, gender, academic achievement, educational track, socioeconomic status (SES), parents’ education, students living arrangements with parents, ethnicity and race, immigrant and minority status, physical appearance and sexual orientation, students’ (academic) behaviour in school (including teacher and parent reports of student behaviour), substance use, social media use, emotional feelings and functioning, students’ self-perception, students’ perceptions of the environment around them, well-being, students’ expectations, social goals, coping strategies, and problem-solving skills.	Age, gender, academic achievement, educational track, students living arrangements with parents, ethnicity and race, immigrant and minority status, physical appearance and sexual orientation, students’ (academic) behaviour in school (including teacher and parent reports of student behaviour), problem behaviour, substance use, social media use, emotional feelings, and functioning, students’ perceptions of the environment around them, internalising difficulties, externalising difficulties, sensation seeking, social goals, coping strategies, and problem-solving skills.
Micro	Parent and family relationships, parent involvement, peer relationships, and relationships with teachers.	Parent and family relationships, parent involvement, problematic peers, discrimination, and being a victim of bullying.
Meso	School size, SES at school level, school composition, teaching practices, classroom goals, classroom climate, school support practices, autonomy supporting practices, school disciplinary practices, other school policies and practices, and extracurricular activities.	School size, SES at school level, school composition, school violence, teaching practices, classroom goals, school disciplinary practices, other school policies and practices, and extracurricular activities.
Exo	Student’s participation in youth-based community organisations, percentage of non-US citizens in the neighbourhood.	Educational instability due to residential changes, the percentage of renters in the neighbourhood, schools in urban areas, and students paying their rent.
Macro	Higher gross domestic product, religiosity, and OECD country.	Hierarchical cultures.
Chrono	High school belonging at previous time points.	Low school belonging in middle school.

**Table 3:** Predictors of the sense of school belonging of different groups of students.

			Findings		
Level	Predictor	Study	Positive Predictors	Negative Predictors	Non-Significant Predictor
Individual	Age	Sampasa-Kanyinga et al. (2019) [43]		Age (for high school students)	Age (for middle school students)
	Academic achievement	Ma (2003) [16]	Academic achievement (for 8th grade students)	Academic achievement (for 6th grade students)	
	Educational track	Aerts et al. (2012) [17]	Arts educational track (for girls)		Arts educational track (for boys)
		Aerts et al. (2012) [17]		Technical track (for boys)	Technical track (for girls)
	SES	Ma (2003) [16]	SES (for 8th grade students)		SES (for 6th grade students)
	Students’ living arrangements	Azagba et al. (2014) [87]		Not living with both parents (for girls)	Not living with both parents (for boys)
	Physical appearance and sexual orientation	Aerts et al. (2012) [17]		Bisexual and homosexual orientation (for girls)	Bisexual and homosexual orientation (for boys)
	Social media use	Sampasa-Kanyinga et al. (2019) [43]	Moderate use of social media (for high school students)	Heavy use of social media (for middle school students)	
	Externalising difficulties	Bao et al. (2018) [60]		Sleep problems (for boys)	Sleep problems (for girls)
Micro	Parent involvement	Uslu and Gizir (2017) [77]	Parental involvement at school (for boys)		Parental involvement at school (for girls)
		Uslu and Gizir (2017) [77]	Parental involvement at home (for girls)		Parental involvement at home (for boys)
	Teaching practices	He and Fischer (2020) [94]	Soft grading (for immigrant students in Italy)	Soft grading (for non-immigrant students in Italy)	
		He and Fischer (2020) [94]		Soft grading (for immigrant students in Germany)	Soft grading (for non-immigrant students in Germany)
		He and Fischer (2020) [94]	Hard grading (for non-immigrant students in Spain)		Hard grading (for immigrant students in Spain)
		He and Fischer (2020) [94]	Hard grading (for non-immigrant students in Italy)		Hard grading (for immigrant students in Italy)
		He and Fischer (2020) [94]		Hard grading (for immigrant students in Germany)	Hard grading (for non-immigrant students in Germany)
Meso	Classroom climate	Ma (2003) [16]	Academic pressure (for 6th grade students)		Academic pressure (for 8th grade students)
	School disciplinary practices	Ma (2003) [16]	Disciplinary climate (for 8th grade students)		Disciplinary climate (for 6th grade students)
	Other school policies	He and Fischer (2020) [94]	School language different than heritage language (for immigrant students in Germany)		School language different than heritage language (for immigrant students in Germany)

Note. In all of the studies, the effect size was measured by the regression coefficient and proved to be significant, except for the study conducted by Uslu and Gizir [77].

## Data Availability

No new data were created. Results are based on existing articles on the topic. Data, code and other materials can be accessed by contacting the authors. The review was not previously registered. A protocol was not prepared.

## Supplementary Materials

The following supporting information can be downloaded at: https://www.mdpi.com/article/10.3390/ejihpe14110190/s1, Table S1: Identified predictors of the sense of school belonging according to Bronfenbrenner’s bioecological model (1986) and characteristics of the studies reviewed.

## References

[1] Maslow A.H. (1958). A Dynamic Theory of Human Motivation. Understanding Human Motivation.

[2] Baumeister R.F., Leary M.R. (1995). The Need to Belong: Desire for Interpersonal Attachments as a Fundamental Human Motivation. Psychol. Bull..

[3] Chhuon V., Wallace T.L. (2014). Creating Connectedness Through Being Known: Fulfilling the Need to Belong in U.S. High Schools. Youth Soc..

[4] Kalil A., Ziol-Guest K.M. (2008). Teacher Support, School Goal Structures, and Teenage Mothers’ School Engagement. Youth Soc..

[5] Goodenow C. (1993). The Psychological Sense of School Membership among Adolescents: Scale Development and Educational Correlates. Psychol. Sch..

[6] Allen K.-A., Kern M.L., Vella-Brodrick D., Hattie J., Waters L. (2018). What Schools Need to Know About Fostering School Belonging: A Meta-Analysis. Educ. Psychol. Rev..

[7] Libbey H.P. (2004). Measuring Student Relationships to School: Attachment, Bonding, Connectedness, and Engagement. J. Sch. Health.

[8] Slaten C.D., Ferguson J.K., Allen K.-A., Brodrick D.-V., Waters L. (2016). School Belonging: A Review of the History, Current Trends, and Future Directions. Educ. Dev. Psychol..

[9] Goodenow C., Grady K.E. (1993). The Relationship of School Belonging and Friends’ Values to Academic Motivation Among Urban Adolescent Students. J. Exp. Educ..

[10] Allen K., Vella-Brodrick D., Waters L. (2016). Fostering School Belonging in Secondary Schools Using a Socio-Ecological Framework. Educ. Dev. Psychol..

[11] Bronfenbrenner U. (1986). Ecology of the Family as a Context for Human Development: Research Perspectives. Dev. Psychol..

[12] Bronfenbrenner U., Morris P.A. (2006). The Bioecological Model of Human Development. Handbook of Child Psychology. Volume One: Theoretical Models of Human Development.

[13] Norgate S. (2017). Atypical Development. An Introduction to Developmental Psychology.

[14] Ham S.-H., Yang K.-E., Cha Y.-K. (2017). Immigrant Integration Policy for Future Generations? A Cross-National Multilevel Analysis of Immigrant-Background Adolescents’ Sense of Belonging at School. Int. J. Intercult. Relat..

[15] Loukas A., Ripperger-Suhler K.G., Herrera D.E. (2012). Examining Competing Models of the Associations among Peer Victimization, Adjustment Problems, and School Connectedness. J. Sch. Psychol..

[16] Ma X. (2003). Sense of Belonging to School: Can Schools Make a Difference?. J. Educ. Res..

[17] Aerts S., Van Houtte M., Dewaele A., Cox N., Vincke J. (2012). Sense of Belonging in Secondary Schools: A Survey of LGB and Heterosexual Students in Flanders. J. Homosex..

[18] Bonny A.E., Britto M.T., Klostermann B.K., Hornung R.W., Slap G.B. (2000). School Disconnectedness: Identifying Adolescents at Risk. Pediatrics.

[19] McNeely C.A., Nonnemaker J.M., Blum R.W. (2002). Promoting School Connectedness: Evidence from the National Longitudinal Study of Adolescent Health. J. Sch. Health.

[20] Waters S., Cross D., Shaw T. (2010). Does the Nature of Schools Matter? An Exploration of Selected School Ecology Factors on Adolescent Perceptions of School Connectedness. Br. J. Educ. Psychol..

[21] Smerdon B.A. (2002). Students’ Perceptions of Membership in Their High Schools. Sociol. Educ..

[22] van Houtte M., van Maele D. (2012). Students’ Sense of Belonging in Technical/Vocational Schools Versus Academic Schools: The Mediating Role of Faculty Trust in Students. Teach. Coll. Rec..

[23] Korpershoek H., Canrinus E.T., Fokkens-Bruinsma M., de Boer H. (2019). The Relationships between School Belonging and Students’ Motivational, Social-Emotional, Behavioural, and Academic Outcomes in Secondary Education: A Meta-Analytic Review. Res. Pap. Educ..

[24] Moyano N., Sánchez-Fuentes M.D.M. (2020). Homophobic Bullying at Schools: A Systematic Review of Research, Prevalence, School-Related Predictors and Consequences. Aggress. Violent Behav..

[25] Raniti M., Rakesh D., Patton G.C., Sawyer S.M. (2022). The Role of School Connectedness in the Prevention of Youth Depression and Anxiety: A Systematic Review with Youth Consultation. BMC Public Health.

[26] Mok S.Y., Martiny S.E., Gleibs I.H., Keller M.M., Froehlich L. (2016). The Relationship between Ethnic Classroom Composition and Turkish-Origin and German Students’ Reading Performance and Sense of Belonging. Front. Psychol..

[27] Allen K.-A., Kern M.L., Rozek C.S., McInerney D.M., Slavich G.M. (2021). Belonging: A Review of Conceptual Issues, an Integrative Framework, and Directions for Future Research. Aust. J. Psychol..

[28] Vaz S., Falkmer M., Ciccarelli M., Passmore A., Parsons R., Tan T., Falkmer T. (2015). The Personal and Contextual Contributors to School Belongingness among Primary School Students. PLoS ONE.

[29] D’hondt F., Van Houtte M., Stevens P.A.J. (2015). How Does Ethnic and Non-Ethnic Victimization by Peers and by Teachers Relate to the School Belongingness of Ethnic Minority Students in Flanders, Belgium? An Explorative Study. Soc. Psychol. Educ..

[30] Hernández M.M., Robins R.W., Widaman K.F., Conger R.D. (2017). Ethnic Pride, Self-Esteem, and School Belonging: A Reciprocal Analysis over Time. Dev. Psychol..

[31] Huyge E., Van Maele D., Van Houtte M. (2015). Does Students’ Machismo Fit in School? Clarifying the Implications of Traditional Gender Role Ideology for School Belonging. Gend. Educ..

[32] Loukas A., Cance J.D., Batanova M. (2016). Trajectories of School Connectedness Across the Middle School Years: Examining the Roles of Adolescents’ Internalizing and Externalizing Problems. Youth Soc..

[33] Shochet I.M., Smyth T., Homel R. (2007). The Impact of Parental Attachment on Adolescent Perception of the School Environment and School Connectedness. Aust. N.Z. J. Fam. Ther..

[34] Allen K.-A., Jamshidi N., Berger E., Reupert A., Wurf G., May F. (2022). Impact of School-Based Interventions for Building School Belonging in Adolescence: A Systematic Review. Educ. Psychol. Rev..

[35] Bowles T., Scull J. (2019). The Centrality of Connectedness: A Conceptual Synthesis of Attending, Belonging, Engaging and Flowing. J. Psychol. Couns. Sch..

[36] Quinn S., Oldmeadow J.A. (2013). Is the *i* Generation a ‘We’ Generation? Social Networking Use among 9- to 13-Year-Olds and Belonging. Br. J. Dev. Psychol..

[37] Bond L., Butler H., Thomas L., Carlin J., Glover S., Bowes G., Patton G. (2007). Social and School Connectedness in Early Secondary School as Predictors of Late Teenage Substance Use, Mental Health, and Academic Outcomes. J. Adolesc. Health.

[38] Tian L., Zhao J., Huebner E.S. (2015). School-related Social Support and Subjective Well-being in School among Adolescents: The Role of Self-system Factors. J. Adolesc..

[39] McMahon S.D., Wernsman J. (2009). The Relation of Classroom Environment and School Belonging to Academic Self-Efficacy among Urban Fourth- and Fifth-Grade Students. Elem. Sch. J..

[40] Tian L., Zhang L., Huebner E.S., Zheng X., Liu W. (2016). The Longitudinal Relationship Between School Belonging and Subjective Well-Being in School Among Elementary School Students. Appl. Res. Qual. Life.

[41] Dai D.Y. (2013). Excellence at the Cost of Social Justice? Negotiating and Balancing Priorities in Gifted Education. Roeper Rev..

[42] Page M.J., McKenzie J.E., Bossuyt P.M., Boutron I., Hoffmann T.C., Mulrow C.D., Shamseer L., Tetzlaff J.M., Akl E.A., Brennan S.E. (2021). The PRISMA 2020 Statement: An Updated Guideline for Reporting Systematic Reviews. Int. J. Surg..

[43] Sampasa-Kanyinga H., Chaput J.-P., Hamilton H.A. (2019). Social Media Use, School Connectedness, and Academic Performance Among Adolescents. J. Prim. Prev..

[44] Mikami A.Y., Ruzek E.A., Hafen C.A., Gregory A., Allen J.P. (2017). Perceptions of Relatedness with Classroom Peers Promote Adolescents’ Behavioral Engagement and Achievement in Secondary School. J. Youth Adolesc..

[45] Oelsner J., Lippold M.A., Greenberg M.T. (2011). Factors Influencing the Development of School Bonding among Middle School Students. J. Early Adolesc..

[46] Resnick M.D., Harris L.J., Blum R.W. (1993). The Impact of Caring and Connectedness on Adolescent Health and Well-Being. J. Paediatr Child Health.

[47] Bardach L., Klassen R.M. (2020). Smart Teachers, Successful Students? A Systematic Review of the Literature on Teachers’ Cognitive Abilities and Teacher Effectiveness. Educ. Res. Rev..

[48] Cohen J. (1988). Statistical Power Analysis for the Behavioral Sciences.

[49] Gowing A., Jackson A.C. (2016). Connecting to School: Exploring Student and Staff Understandings of Connectedness to School and the Factors Associated with This Process. Educ. Dev. Psychol..

[50] Gregory A., Hastings R.P., Kovshoff H. (2020). Academic Self-Concept and Sense of School Belonging of Adolescent Siblings of Autistic Children. Res. Dev. Disabil..

[51] Svavarsdottir E.K. (2008). Connectedness, Belonging and Feelings about School among Healthy and Chronically Ill Icelandic Schoolchildren. Scand. J. Caring Sci..

[52] Thompson D.R., Iachan R., Overpeck M., Ross J.G., Gross L.A. (2006). School Connectedness in the Health Behavior in School-Aged Children Study: The Role of Student, School, and School Neighborhood Characteristics. J. Sch. Health.

[53] Williams L.J., Downing J.E. (1998). Membership and Belonging in Inclusive Classrooms: What Do Middle School Students Have to Say?. J. Assoc. Pers. Sev. Handicap..

[54] Bolland K.A., Bolland A.C., Bolland J.M., Church W.T., Hooper L.M., Jaggers J.W., Tomek S. (2016). TRAJECTORIES OF SCHOOL AND COMMUNITY CONNECTEDNESS IN ADOLESCENCE BY GENDER AND DELINQUENT BEHAVIOR: Trajectories of School and Community Connectedness. J. Community Psychol..

[55] Loukas A., Ripperger-Suhler K.G., Horton K.D. (2009). Examining Temporal Associations Between School Connectedness and Early Adolescent Adjustment. J. Youth Adolesc..

[56] Allen K.-A., Gallo Cordoba B., Ryan T., Arslan G., Slaten C.D., Ferguson J.K., Bozoglan B., Abdollahi A., Vella-Brodrick D. (2022). Examining Predictors of School Belonging Using a Socio-Ecological Perspective. J. Child Fam. Stud..

[57] Allen K.-A., Fortune K.C., Arslan G. (2021). Testing the Social-Ecological Factors of School Belonging in Native-Born, First-Generation, and Second-Generation Australian Students: A Comparison Study. Soc. Psychol. Educ..

[58] Lester L., Waters S., Cross D. (2013). The Relationship Between School Connectedness and Mental Health During the Transition to Secondary School: A Path Analysis. Aust. J. Guid. Couns..

[59] Kelly A.B., O’Flaherty M., Toumbourou J.W., Homel R., Patton G.C., White A., Williams J. (2012). The Influence of Families on Early Adolescent School Connectedness: Evidence That This Association Varies with Adolescent Involvement in Peer Drinking Networks. J. Abnorm Child Psychol..

[60] Bao Z., Chen C., Zhang W., Jiang Y., Zhu J., Lai X. (2018). School Connectedness and Chinese Adolescents’ Sleep Problems: A Cross-Lagged Panel Analysis. J. Sch. Health.

[61] Atabey N. (2020). Future Expectations and Self-Efficacy of High School Students as a Predictor of Sense of School Belonging. Educ. Sci..

[62] Datu J.A.D., Valdez J.P.M. (2019). Psychological Capital Is Associated with Higher Levels of Life Satisfaction and School Belongingness. Sch. Psychol. Int..

[63] McDiarmid S., Osman F., Sarkadi A., Durbeej N. (2023). Associations between Social Factors and School Belonging among Newcomer and Non-Newcomer Youth in Sweden. PLoS ONE.

[64] Meisel S.N., Colder C.R. (2017). Social Goals Impact Adolescent Substance Use through Influencing Adolescents’ Connectedness to Their Schools. J. Youth Adolesc..

[65] Mouratidis A.A., Sideridis G.D. (2009). On Social Achievement Goals: Their Relations with Peer Acceptance, Classroom Belongingness, and Perceptions of Loneliness. J. Exp. Educ..

[66] Ahmadi S., Hassani M., Ahmadi F. (2020). Student- and School-Level Factors Related to School Belongingness among High School Students. Int. J. Adolesc. Youth.

[67] Allen K.-A., Cordoba B.G., Parks A., Arslan G. (2022). Does Socioeconomic Status Moderate the Relationship Between School Belonging and School-Related Factors in Australia?. Child Ind. Res..

[68] Booker K.C., Lim J.H. (2018). Belongingness and Pedagogy: Engaging African American Girls in Middle School Mathematics. Youth Soc..

[69] Chiu M.M., Chow B.W.-Y., McBride C., Mol S.T. (2016). Students’ Sense of Belonging at School in 41 Countries: Cross-Cultural Variability. J. Cross-Cult. Psychol..

[70] Dukynaite R., Dudaite J. (2017). Influence of School Factors on Students’ Sense of School Belonging. New Educ. Rev..

[71] Faircloth B.S. (2009). Making the Most of Adolescence: Harnessing the Search for Identity to Understand Classroom Belonging. J. Adolesc. Res..

[72] Froiland J.M., Davison M.L., Worrell F.C. (2016). Aloha Teachers: Teacher Autonomy Support Promotes Native Hawaiian and Pacific Islander Students’ Motivation, School Belonging, Course-Taking and Math Achievement. Soc. Psychol. Educ..

[73] Golaszewski N.M., Pasch K.E., Fernandez A., Poulos N.S., Batanova M., Loukas A. (2018). Perceived Weight Discrimination and School Connectedness Among Youth: Does Teacher Support Play a Protective Role?. J. Sch. Health.

[74] Keyes T.S. (2019). A Qualitative Inquiry: Factors That Promote Classroom Belonging and Engagement Among High School Students. Sch. Community J..

[75] Maurizi L.K., Ceballo R., Epstein-Ngo Q., Cortina K.S. (2013). Does Neighborhood Belonging Matter? Examining School and Neighborhood Belonging as Protective Factors for Latino Adolescents. Am. J. Orthopsychiatry.

[76] Ullman J. (2022). Trans/Gender-Diverse Students’ Perceptions of Positive School Climate and Teacher Concern as Factors in School Belonging: Results from an Australian National Study. Teach. Coll. Rec. Voice Scholarsh. Educ..

[77] Uslu F., Gizir S. (2017). School Belonging of Adolescents: The Role of Teacher–Student Relationships, Peer Relationships and Family Involvement. Educ. Sci.-Theory Pract..

[78] Kashy-Rosenbaum G., Aizenkot D. (2020). Exposure to Cyberbullying in WhatsApp Classmates‘ Groups and Classroom Climate as Predictors of Students‘ Sense of Belonging: A Multi-Level Analysis of Elementary, Middle and High Schools. Child. Youth Serv. Rev..

[79] Craggs H., Kelly C. (2017). School Belonging: Listening to the Voices of Secondary School Students Who Have Undergone Managed Moves. Sch. Psychol. Int..

[80] Liu Y., Kim H., Carney J.V., Chung K.S., Hazler R.J. (2020). Individual and Contextual Factors Associated with School Connectedness in the Context of Counseling in Schools. J. Couns. Dev..

[81] Yuen M., Lau P.S.Y., Lee Q.A.Y., Gysbers N.C., Chan R.M.C., Fong R.W., Chung Y.B., Shea P.M.K. (2012). Factors Influencing School Connectedness: Chinese Adolescents’ Perspectives. Asia Pac. Educ. Rev..

[82] Anderman E.M. (2002). School Effects on Psychological Outcomes during Adolescence. J. Educ. Psychol..

[83] Cueto S., Guerrero G., Sugimaru C., Zevallos A.M. (2010). Sense of Belonging and Transition to High Schools in Peru. Int. J. Educ. Dev..

[84] Johnson R.M., Strayhorn T.L., Parler B. (2020). “I Just Want to Be a Regular Kid:” A Qualitative Study of Sense of Belonging among High School Youth in Foster Care. Child. Youth Serv. Rev..

[85] Lardier D.T., Opara I., Bergeson C., Herrera A., Garcia-Reid P., Reid R.J. (2019). A Study of Psychological Sense of Community as a Mediator between Supportive Social Systems, School Belongingness, and Outcome Behaviors among Urban High School Students of Color. J. Community Psychol..

[86] McInerney K. (2022). Perceptions from Newcomer Multilingual Adolescents: Predictors and Experiences of Sense of Belonging in High School. Child Youth Care Forum.

[87] Azagba S., Asbridge M., Langille D.B. (2014). Is Religiosity Positively Associated with School Connectedness: Evidence from High School Students in Atlantic Canada?. J. Primary Prevent.

[88] Fulginiti A., He A.S., Negriff S. (2018). Suicidal Because I Don’t Feel Connected or Vice Versa? A Longitudinal Study of Suicidal Ideation and Connectedness among Child Welfare Youth. Child Abus. Negl..

[89] Karcher M.J. (2005). The Effects of Developmental Mentoring and High School Mentors’ Attendance on Their Younger Mentees’ Self-Esteem, Social Skills, and Connectedness. Psychol. Schs..

[90] Mrug S., Windle M. (2009). Bidirectional Influences of Violence Exposure and Adjustment in Early Adolescence: Externalizing Behaviors and School Connectedness. J. Abnorm Child Psychol..

[91] Nickerson A.B., Hopson L.M., Steinke C.M. (2011). School Connectedness in Community and Residential Treatment Schools: The Influence of Gender, Grades, and Engagement in Treatment. Child. Youth Serv. Rev..

[92] Norwalk K.E., Hamm J.V., Farmer T.W., Barnes K.L. (2016). Improving the School Context of Early Adolescence Through Teacher Attunement to Victimization: Effects on School Belonging. J. Early Adolesc..

[93] Shochet I.M., Dadds M.R., Ham D., Montague R. (2006). School Connectedness Is an Underemphasized Parameter in Adolescent Mental Health: Results of a Community Prediction Study. J. Clin. Child Adolesc. Psychol..

[94] He J., Fischer J. (2020). Differential Associations of School Practices with Achievement and Sense of Belonging of Immigrant and Non-Immigrant Students. J. Appl. Dev. Psychol..

[95] OECD (2017). PISA 2015 Results (Volume III): Students’ Well-Being.

[96] Berryman M., Eley E. (2019). Student Belonging: Critical Relationships and Responsibilities. Int. J. Incl. Educ..

[97] Cornelius-White J. (2007). Learner-Centered Teacher-Student Relationships Are Effective: A Meta-Analysis. Rev. Educ. Res..

[98] DeNicolo C.P., Yu M., Crowley C.B., Gabel S.L. (2017). Reimagining Critical Care and Problematizing Sense of School Belonging as a Response to Inequality for Immigrants and Children of Immigrants. Rev. Res. Educ..

[99] Allen K.-A., Kern P. (2019). Boosting School Belonging. Practical Strategies to Help Adolescents Feel Like They Belong at School.

[100] Allen K.-A., Berger E., Reupert A., Grove C., May F., Patlamazoglou L., Gamble N., Wurf G., Warton W. (2023). Student-Identified Practices for Improving Belonging in Australian Secondary Schools: Moving Beyond COVID-19. Sch. Ment. Health.

[101] Shochet I.M., Smith C.L., Furlong M.J., Homel R. (2011). A Prospective Study Investigating the Impact of School Belonging Factors on Negative Affect in Adolescents. J. Clin. Child Adolesc. Psychol..

[102] Kuttner P.J. (2023). The Right to Belong in School: A Critical, Transdisciplinary Conceptualization of School Belonging. AERA Open.

