# Supplementary material for: Addressing the Sense of School Belonging Among All Students? A Systematic Literature Review
Source: Eur J Investig Health Psychol Educ. 2024 Nov 12;14(11):2901–17. doi: 10.3390/ejihpe14110190 (PMC11592540; doi:10.3390/ejihpe14110190)
Supplement: Supplementary file 1 [file ejihpe-14-00190-s001.zip › ejihpe-3250663-supplementary.pdf]

Table S1. Identified predictors of the sense of school belonging according to Bronfenbrenner's bioecological model (1986) and characteristics of the studies reviewed.

				Findings			
**Level**	**Predictor**	**Studies not confirming the association**	**Studies confirming the association**	**Positive predictors**	**Effect size**	**Negative predictors**	**Effect size**
**Individual**	Age	8 (Aerts et al., 2012; Dotterer et al., 2009; Fulginiti et al., 2018; Golaszewski et al., 2018; Gowing & Jackson, 2016; Gregory et al., 2020; McDiarmid et al 2023; Ullman et al., 2022)	16 (Anderman, 2002; Bolland et al., 2016; Carney et al., 2019; Celeste et al., 2019; Datu & Valdez, 2019; Day et al., 2016; Kashy-Rosenbaum & Aizenkot, 2020; Kelly et al., 2012; Liu et al., 2020; Marino et al., 2020; McNeely et al., 2002; Mrug & Windle, 2009; Sampasa-Kanyinga et al., 2019; Thompson et al., 2006; van Houtte & van Maele, 2012; Vaquera, 2009)	Age	Trivial to small effect (correlation); studies mostly reported significant regression coefficient	Age	Small effect (correlation); studies mostly reported significant regression coefficient

	Gender	14 (Cupito et al., 2015; d'Hondt et al., 2015; Datu & Valdez; 2019; Dotterer et al., 2009; Fulginiti et al., 2018; Gregory et al., 2020; Ham et al., 2017; Liu et al., 2020; Marino et al., 2020; McDiarmid et al., 2023; Rostosky et al., 2003; Sampasa-Kanyinga et al., 2019; van Houtte & van Maele, 2012; Waters et al., 2010)	24 (Allen et al., 2021; Al Yagon et al., 2016; Azagba et al., 2014; Bolland et al., 2016; Carney et al., 2019; Day et al., 2016; Delgado et al., 2016; Golaszewski et al., 2018; He & Fischer, 2020; Huyge et al., 2015; Kashy-Rosenbaum & Aizenkot, 2020; Kelly et al., 2012; Loukas et al., 2016; Ma, 2003; McInerney, 2022; McNeely et al., 2002; Mikami et al., 2017; Nickerson et al., 2011; Oelsner et al., 2011; Resh & Sabbagh, 2014; Smerdon, 2002; Thompson et al., 2006; Vaquera, 2009; Vaz et al., 2015)	Female students tend to have a greater sense of belonging	Trivial to small effect (correlation); studies mostly reported significant regression coefficient	Female students tend to have a lower sense of belonging	Small effect (correlation); studies mostly reported significant regression coefficient
	Academic achievement	3 (d'Hondt et al., 2015; McInerney, 2022; Mok et al., 2016)	14 (Azagba et al., 2014; E.M. Anderman, 2002; L.H. Anderman, 2003; Chiu et al., 2016; Cueto et al., 2010; Ma, 2003; McNeely et al., 2002; Nickerson et al., 2011; Oelsner et al., 2011; Rostosky et al., 2003; Smerdon, 2002; Thompson et al., 2006; van Houtte & van Maele, 2012; Waters et al., 2010)	Higher academic achievement	Small to moderate effect (correlation), studies mostly reported significant regression coefficient	Lower academic achievement	Studies mostly reported significant regression coefficient

Educational track	2 (Huyge et al., 2015; van Houtte & van Maele, 2012)	4 (Aerts et al., 2012; Celeste et al., 2019; d'Hondt et al., 2015; Smerdon, 2002)	Arts vocational track (female students)	Significant regression coefficient	Being in a non-academic educational track, technical, or vocational school	Significant regression coefficients
Socioeconomic status (SES)	5 (d'Hondt et al., 2015; Huyge et al., 2015; McDiarmid et al., 2023; Smerdon, 2002; Vaz et al., 2015)	13 (Allen, Cordoba et al., 2022; Allen, Cordoba, Parks et al., 2022; Allen et al., 2021; Ahmadi et al., 2020; Azagba et al., 2014; Chiu et al., 2016; Cueto et al., 2010; He & Fischer, 2020; Ma, 2003; Mok et al., 2016; Mrug & Windle, 2009; Sampasa-Kanyinga et al., 2019; van Houtte & van Maele, 2012)	Students' SES, family income or wealth, ESCS	Small effect (correlation); studies mostly reported significant regression coefficients		
Parents' education	4 (Azagba et al., 2014; Kelly et al., 2012; Resh & Sabbagh; 2014; Thompson et al., 2014)	7 (Anderman, 2002; Chiu et al., 2016; Dotterer et al., 2009; Ham et al., 2017; Marksteiner & Kruger, 2016; McInerney, 2022; Vaquera, 2009)	Higher educational level, number of books at home, father's education	Small effect (correlation); studies mostly report significant regression coefficients		
Students' living arrangements with parents	2 (Ma, 2003; McInerney, 2022)	3 (Azagba et al., 2014; McNeely et al., 2002; Thompson et al., 2006)	Living with both parents in the same household	Significant regression coefficient	Not living with both parents	Significant regression coefficient
Ethnicity and race	3 (Fulginiti et al., 2018; Liu et al., 2020; Mok et al., 2016)	7 (E.M. Anderman, 2002; Day et al., 2016; Golaszewski et al., 2018; McNeely et al., 2002; Mikami et al., 2017; Sampasa-Kanyinga et al., 2019; Vaquera, 2009)	White, Black, or Latino (Mexican, Central/South)	Significant regression coefficients	African American, Hispanic, American Indian, Native American, Black, Hawaiian, Asian, or Multiple ethnicities	Significant regression coefficients

Immigrant and minority status	6 (Huyge et al., 2015; Ma, 2003; McInerney, 2022; Mok et al., 2016; Ullman et al., 2022; van Houtte & van Maele, 2012)	6 (Celeste et al., 2019; Chiu et al., 2016; Kelly et al., 2012; Ham et al., 2017; Smerdon, 2002; Vaquera, 2009)	Speaking a language other than English at home, minority status, or being a first-generation immigrant	Significant regression coefficients	Immigrant background, ethnic minority status	Small effect (correlation); studies mostly reported significant regression coefficients
Physical appearance and sexual orientation	1 (Karcher, 2005)	4 (Aerts et al., 2012; Rostosky et al., 2003; Thompson et al., 2006; Vaz et al., 2015)	Perception of being good-looking, physical appearance competence	Significant regression coefficients	Sexual minority status, sexual orientation	Trivial to small effect (correlation); studies mostly reported significant regression coefficients
Students' (academic) behaviour in school, including teacher and parent reports of student behaviour	2 (Cueto et al., 2010; Karcher, 2005)	8 (Gregory et al., 2020; Gowing & Jackson, 2016; McNeely et al., 2002; Smerdon, 2002; Svavarsdottir, 2009; Waters et al., 2010; Williams & Downing, 1998; Thompson et al., 2006)	Active participation in doing homework, academic engagement, prosocial behaviour, number of math and English courses, teacher reports of behaviour	Small to moderate effect (correlation); studies mostly reported significant regression coefficients	Negative: skipping school, exhibiting negative academic behaviour, externalizing difficulties (parents), behaviour dysregulation (teachers)	Studies reported significant regression coefficients
Problem behaviour	1 (McDiarmid et al., 2023)	3 (Bolland et al., 2016; Loukas et al., 2009; Oelsner et al., 2011;			Deviant behaviour, delinquency, and conduct problems	Moderate effect (correlation); studies mostly reported significant regression coefficients
Substance use	1 (Fulginiti et al., 2018)	6 (Azagba et al., 2014; Kelly et al., 2012; Gowing & Jackson, 2016; Meisel & Colder, 2017; Oelsner et al., 2011; Sampasa-Kanyinga et al., 2019)	Substance use, cigarette use	Small effect (correlation); significant regression coefficients	Substance use, cigarette use, alcohol use	Small effect (correlation); studies mostly reported significant regression coefficients
Social media use		1 (Sampasa-Kanyinga et al., 2019)	Moderate use of social media	Significant regression coefficient	Heavy use of social media (middle school)	Significant regression coefficient

	Emotional functioning and feelings		10 (Allen, Cordoba et al., 2022; Allen et al., 2021; Anderman, 2003; Craggs & Kelly, 2017; Frydenberg et al., 2009; McDiarmid et al., 2023; Ritchie & Gaulter, 2020; Waters et al., 2010; Williams & Downing, 1998; Zhao & Zhao, 2015)	Emotional well-being, cognitive reappraisal, enjoyment, feeling safe, academic task values, not having a difficult transition in primary school, achievement motivation	Moderate effect (correlation), studies mostly reported significant regression coefficients	Emotional symptoms, expressive suppression	Trivial to moderate effect (correlation); significant regression coefficients
	Students' self-perception	1 (Karcher, 2005)	7 (Anderman, 2002; Atabey, 2020; Chiu et al., 2016; Datu & Valdez; 2017; Hernandez et al., 2017; Ma, 2003; Resh & Sabbagh, 2014)	Self-efficacy, self-esteem, (academic) self-concept, academic self-image, psychological capital	Moderate to large effect (correlation): studies mostly reported significant regression coefficients		
	Students' perceptions of the environment around them		8 (Ahmadi, et al., 2020; Allen, Cordoba et al., 2022; Allen et al., 2021; Carney et al., 2019; Lardier et al., 2019; Huyge et al., 2015; Hernandez et al., 2017; Marksteiner & Kruger, 2016)	Sense of being a team, sense of fairness, psychological sense of community, ethnic pride, enjoyment of cooperation, value of cooperation	Small to moderate effect (correlation), studies mostly reported significant regression coefficients	Beliefs about traditional gender roles	Significant regression coefficient
	Internalizing difficulties	3 (Fulginiti et al., 2018; Loukas et al., 2009; Shochet et al., 2006)	7 (Allen, Cordoba et al., 2022; Allen et al., 2021; Gregory et al., 2020; Kelly et al., 2012; Lester et al., 2013; Loukas et al., 2016; Meisel & Colder, 2017)			Internalizing difficulties and symptoms, depression, anxiety, test anxiety	Small to moderate effect (correlations), significant regression coefficients

	Externalizing difficulties		3 (Bao et al., 2018; Gregory et al., 2020; Loukas et al., 2016)			Externalizing difficulties; sleep problems	Small to moderate effect (correlations), significant regression coefficients
	Well-being	2 (Gowing & Jackson, 2016; Shochet et al., 2006)	4 (Datu & Valdez, 2019; Ma, 2003; McDiarmid et al., 2023; Tian et al., 2016)	General health, school satisfaction, affect in school, life satisfaction, prosocial behaviour	Moderate to large effect (correlations); significant regression coefficients		
	Students' expectations	1 (Anderman, 2003)	2 (Atabey, 2020; Smerdon, 2002)	Future and educational expectations	Moderate effect (correlation), significant regression coefficients		
	Sensation seeking		2 (Azagba et al., 2014; Kelly et al., 2012)			Sensation seeking	Small effect (correlation); significant regression coefficients
	Social goals		3 (Meisel & Colder, 2017; Mouratidis & Sideridis, 2009; Vaz et al., 2015)	Social affiliation goals for school, social development goals	Large effect (correlation), significant regression coefficients	Agentic goals	Small effect (correlation); significant regression coefficient
	Coping strategies and problem-solving skills		2 (Frydenberg et al., 2009; Vaz et al., 2015)	Productive coping strategies	Significant regression coefficient	Non-productive coping strategies and problem-solving skills	Significant regression coefficients

Micro	Parent and family relationships	1 (Ma, 2003)	9 (Al-Yagon et al., 2016; Chiu et al., 2016; Cupito et al., 2015; Fulginiti et al., 2018; Gregory et al., 2020; Kelly et al., 2012; van Houtte & van Maele, 2012; Shochet et al., 2007; Waters et al., 2010)	Connectedness to family, caregiver connectedness, attachment to mother, family relationship quality, parental support, family social communication, parental adolescent attachment, familism, filial obligation	Small to moderate effect (correlation), studies mostly reported significant regression coefficients	Sibling with autism	Significant regression coefficient
	Parent involvement	1 (Ma, 2003)	6 (Ahmadi et al., 2020; Allen, Cordoba et al., 2022; Allen et al., 2021; Thompson et al., 2006; Uslu & Gizir, 2017; Vaz et al., 2015)	Involvement at school or home, parents willing to help with homework or talk with teachers, parents expecting a university degree, parental emotional support	Moderate effect (correlation), significant regression coefficients	Low school-based involvement of parents	Significant regression coefficient
	Peer relationships	2 (McDiarmid et al., 2023; Waters et al., 2010)	15 (Ahmadi et al., 2020; Cemalcilar, 2010; Craggs & Kelly, 2017; Delgado et al., 2016; Faircloth, 2009; Gowing, 2019; Liu et al., 2020; Maurizi et al., 2013; Meisel & Colder, 2017; Ritchie & Gaulter, 2018; Thompson et al., 2006; Uslu & Gizir, 2017; Vaquera, 2009; Williams & Downing, 1998; Yuen et al., 2012)	Peer support, peer relationships, friendship quality and perceived number of friends, social skills	Small to moderate effect (correlation), studies mostly reported significant regression coefficients		

	Problematic peers	3 (Delgado et al., 2016; Kelly et al., 2012; Oelsner et al., 2011)		Antisocial peers, peer drinking networks, friend problem behaviour	Small effect (correlation), significant regression coefficients
	Discrimination	4 (Aerts et al., 2012; Dotterer et al., 2009; Golaszewski et al., 2018; McDiarmid et al., 2023)		Discrimination in school setting, by peers, by teachers, weight discrimination by peers, perceived ethnic discrimination	Small effect (correlation), studies mostly reported significant regression coefficients
	Being a victim of bullying	8 (Allen, Cordoba, Parks et al., 2022; Day et al., 2016; d'Hondt et al., 2015; Hatchel et al., 2019; Kashy-Rosenbaum & Aizenkot, 2020; Loukas et al., 2012; Mrug & Windle, 2009; Vaz et al., 2015)		Relational victimization, experiencing cyberbullying as victims, non-ethnic victimization by peers and teachers, ethnic victimization by peers and teachers, experiencing homophobic bullying, being bullied in primary school, witnessing violence	Small to moderate effect (correlation), significant regression coefficients

	Relationships with teachers		15 (Ahmadi et al., 2020; Allen, Cordoba, Parks et al., 2022; Booker & Lim, 2018; Chiu et al., 2016; Dukynaite & Dudaite, 2017; Faircloth, 2009; Froiland et al., 2016; Golaszewski et al., 2018; Keyes, 2019; Maurizi et al., 2013; Shochet et al., 2007; Ullman et al., 2022; Uslu & Gizir, 2017; Waters et al., 2010; Williams & Downing, 1998)	Relationship with teachers, connectedness to teachers, teachers support, teacher likeability, teacher concern	Moderate to large effect (correlations); significant regression coefficients		
**Meso**	School size	5 (Anderman, 2002; d'Hondt et al., 2015; Ham et al., 2017; Ma, 2003; Vaquera, 2009)	4 (Day et al., 2016; McNeely et al., 2002; Thompson et al., 2006; Waters et al., 2010)	Bigger schools	Small effect (correlation), significant regression coefficient	Bigger schools	Significant regression coefficient
	SES at the school level	1 (Ma, 2003)	8 (Chiu et al., 2016; Day et al., 2016; Ham et al., 2017; He & Fischer, 2020; Huyge et al., 2015; Smerdon, 2002; Thompson et al., 2006; Vaquera, 2009)	Higher SES of school, SES homogeneity of schoolmates	Significant regression coefficients	Higher SES of school, Lower SES of school	Significant regression coefficients
	School composition	4 (d'Hondt et al., 2015; Ham et al., 2017; Ullman et al., 2022; van Houtte & van Maele, 2012)	4 (Celeste et al., 2019; Huyge et al., 2015; McNeely et al., 2002; Mok et al., 2016)	Higher ethnic composition	Significant regression coefficient	Percentage of female students in school, higher ethnic composition	Significant regression coefficients
	School violence		2 (Cemalcilar, 2010; Kashy-Rosenbaum & Aizenkot, 2020)			Perceived violence in the school environment, reports of cyberbullying	Moderate effect (correlation); significant regression coefficients

	Teaching practices		10 (Alesech & Nayar, 2019; Allen, Cordoba et al., 2022; Allen et al., 2021; Booker & Lim, 2018; Day et al., 2016; He & Fischer, 2020; Keyes, 2019; Ullman et al., 2022; Vaz et al., 2015; Williams & Downing, 1998)	Student participation, inclusive school environment, supportive practices by teachers towards students, showing respect, being appreciative of students’ work, treating students equally, grading practices, high expectations of teachers for success	Significant regression coefficients	Showing favouritism towards certain students, extending low invitations for parental involvement in students' schooling, grading practices, teacher unfairness	Significant regression coefficients
	Classroom goals		2 (Vaz et al., 2015; Walker, 2012)	Classroom-mastery goals	Moderate effect (correlation), significant regression coefficient	Classroom performance-avoidance goals, low level of task goal structure	Small effect (correlation), significant regression coefficients
	Classroom climate	1 (Shochet et al., 2007)	4 (Kashy-Rosenbaum & Aizenkot, 2020; Ma, 2003; McNeely et al., 2002; Waters et al., 2010)	Positive classroom climate	Moderate to large effect (correlation); significant regression coefficients		
	School support practices		5 (Craggs & Kelly, 2017; Liu et al., 2020; Shochet et al., 2007; Yuen et al., 2012; Waters et al., 2010)	School guidance and support services, identification and support of special educational needs, priority for pastoral care, school support for learning	Large effect (correlation), studies mostly reported significant regression coefficients		

	Autonomy-supporting practices	1 (Ahmadi et al., 2020)	5 (Gowing & Jackson, 2016; Smerdon, 2002; van Houtte & van Maele, 2012; Vaz et al., 2015; Yuen et al., 2012)	Students' authority over academic work, student voice, student involvement in classroom activities, providing opportunities for achievement and leadership, faculty trust in students	Small effect (correlation); studies mostly report significant regression coefficients		
	School disciplinary practices		6 (Allen, Cordoba et al., 2022; Allen et al., 2021; Chiu et al., 2016; Ma, 2003; McNeely et al., 2002; Yuen et al., 2012)	Higher disciplinary climate, disciplinary climate in science classes	Significant regression coefficients	Harsh or modal disciplinary climate	Significant regression coefficients
	Other school policies and practices		7 (Aerts et al., 2012; Anderman, 2002; Celeste et al., 2019; He & Fischer, 2020; Liu et al., 2020; Smerdon, 2002; Ullman et al., 2022)	Homeroom, perceived school friendliness towards LGB, acceptance of diversity, school's acceptance and support of gender and sexuality diversity	Significant regression coefficients	Busing practices, assimilationism, grouping students in classes based on subjects or ability, assimilationism practices (minority students)	Significant regression coefficients
	Extracurricular activities	1 (Maurizi et al., 2013)	7 (Craggs & Kelly, 2017; Gowing & Jackson, 2016; McNeely et al., 2002; Shochet et al., 2007; Thompson et al., 2006; Waters et al., 2010; Yuen et al., 2012)	Participation in extracurricular activities, talent development opportunities	Small to moderate effect (correlation), significant regression coefficients	Not participating in extracurricular activities	Significant regression coefficient

**Exo**		7 (Gregory et al., 2020; Huyge et al., 2015; McNeely et al., 2002; Oelsner et al., 2011; Ullman et al., 2022; Vaquera, 2009; van Houtte & van Maele, 2012)	6 (Anderman, 2002; Cueto et al., 2010; Johnson et al., 2020; Lardier et al., 2019; McInerney, 2022; Thompson et al., 2006)	Participation in youth-based community organizations, percentage of non-US citizens in the neighbourhood	Significant regression coefficients	Educational instability due to residential changes, percentage of renters in the neighbourhood, schools in urban areas, students paying their rent	Significant regression coefficients
**Macro**			4 (Allen, Cordoba et al., 2022; Azagba et al., 2014; Chiu et al., 2016; Ham et al., 2017)	Higher GDP, religiosity, OECD country	Significant regression coefficients	Hierarchical cultures	Significant regression coefficient
**Chrono**			11 (Bao et al., 2018; Fulginiti et al., 2018; Karcher, 2005; Loukas et al., 2009; Loukas et al., 2012; Mikami et al., 2017; Mrug & Windle, 2009; Nickerson et al., 2011; Norwalk et al., 2016; Shochet et al., 2006; Smerdon, 2002)	High school belonging at previous time points	Moderate to large effect (correlations); significant regression coefficient	Low school belonging in middle school	Significant regression coefficient
